# Atomically dispersed hybrid nickel-iridium sites for photoelectrocatalysis

**DOI:** 10.1038/s41467-017-01545-w

**Published:** 2017-11-07

**Authors:** Chunhua Cui, Marc Heggen, Wolf-Dietrich Zabka, Wei Cui, Jürg Osterwalder, Benjamin Probst, Roger Alberto

**Affiliations:** 1 Department of Chemistry, University of Zürich, Winterthurerstrasse 190, CH-8057 Zürich, Switzerland; 2 Institute of Fundamental and Frontier Sciences, University of Electronic Science and Technology of China, Chengdu, 610054 China; 3 Ernst Ruska-Centre for Microscopy and Spectroscopy with Electrons, Forschungszentrum Juelich GmbH, Juelich, 52425 Germany; 4 Department of Physics, University of Zürich, Winterthurerstrasse 190, CH-8057 Zürich, Switzerland

## Abstract

Atomically dispersed supported catalysts can maximize atom efficiency and minimize cost. In spite of much progress in gas-phase catalysis, applying such catalysts in the field of renewable energy coupled with electrochemistry remains a challenge due to their limited durability in electrolyte. Here, we report a robust and atomically dispersed hybrid catalyst formed in situ on a hematite semiconductor support during photoelectrochemical oxygen evolution by electrostatic adsorption of soluble monomeric [Ir(OH)_6_]^2−^ coupled to positively charged NiO_x_ sites. The alkali-stable [Ir(OH)_6_]^2−^ features synergistically enhanced activity toward water oxidation through NiO_x_ that acts as a “movable bridge” of charge transfer from the hematite surface to the single iridium center. This hybrid catalyst sustains high performance and stability in alkaline electrolyte for >80 h of operation. Our findings provide a promising path for soluble catalysts that are weakly and reversibly bound to semiconductor-supported hole-accumulation inorganic materials under catalytic reaction conditions as hybrid active sites for photoelectrocatalysis.

## Abstract (web summary)

Atomically disperse catalysts can offer promising activity due to the high exposure of active sites. Here, iridium complexes in solution undergo a binding equilibrium with a nickel oxide surface resulting in atomically disperse iridium and high turnover frequencies for oxygen evolution.

## Introduction

During the past decade, atomically dispersed supported catalysts with 100% exposure of catalytic sites to reactants have attracted increasing research interest and triggered industrial applications^1,2^, but are rarely exploited for electrocatalysis at solid–liquid interfaces owing to their limited stability^3–6^. One of the harshest reactions in converting solar energy into chemical fuels is the photoelectrochemical oxidation of water to oxygen^7^. This catalytic process needs a bi-component device with a semiconductor photoabsorber that transfers holes to a robust catalyst for oxidizing water^8,9^. However, there has been limited success in immobilizing atomic-scale inorganic materials and/or molecular complexes on cheap semiconductor supports for a long-term reaction, especially in alkaline electrolyte with a reduced energy loss for the oxygen evolution reaction (OER)^10,11^. Precious metal oxides, such as IrO_x_, are superior OER catalysts but their cluster-type or monolayer structures are prone to hydroxylation and thus become soluble/detachable in alkaline electrolytes^12–16^. For highly active molecular catalysts grafted on semiconductor supports, the practical operating potential required to drive OER can cleave the organic ligands and lead to the formation of deactivated metal oxide nanoparticles or soluble metal hydroxide species^17,18^. Thus, immobilizing a soluble catalyst directly under OER conditions might present an attractive solution, but some critical issues still remain unresolved^19–22^—how to bridge the communication between soluble catalyst species and semiconductor surfaces, and how to stabilize the performance and integrity of such a system over an extended time scale.

Here, we report on a hybrid OER catalyst that contains supported sublayer MO_x_ sites (M = Fe, Cu, Co, Ni, and Ti) and soluble monomeric hexahydroxyiridate (IV) [Ir(OH)_6_]^2−^ anions (Ir-anions). As an ideal molecular model, we uncover a close relationship between the hole accumulation capacity of MO_x_ and the enhanced activity of Ir-anions. Among the MO_x_ sites, the NiO_x_ showed the highest capacity of hole accumulation and favorable weak “Fe/Ni–O–Ir” bonds with Ir-anions during OER, allowing the accumulated holes to subsequently transfer to Ir sites for OER. Contrary to the prevailing notion that the redox species in an electrolyte acts as hole scavenger, thus poisoning the OER^23,24^, we observe that the Ir-anion coupled to hematite supported NiO_x_ (H-NiO_x_) demonstrates unusual OER activity enhancement with a turnover frequency of 2.4–12.7 s^−1^ per Ir site at 1.23 *V*_RHE_ (Supplementary Note 1). This hybrid catalyst demonstrates superior stability in strong alkaline electrolytes (even 4.0 M NaOH) for >80 h. It combines the advantages of soluble molecular catalysts with fully exposed active sites and the benefits of solid-state materials with unique physicochemical properties, bridging the gap between heterogeneous and homogeneous catalysts.

## Results

### Catalyst loading

Nanostructured hematite was applied as a support for catalyst loading (Supplementary Fig. 1). In order to avoid the influence of foreign metals on the surface properties, no extrinsic doping was intentionally introduced into the hematite (Supplementary Note 2). The MO_x_ was loaded onto the hematite through a facile transient electroreduction of metal cations to metallic sites (Supplementary Fig. 2) (see Methods section). Controlling the charge going through the circuit manipulates the amount of metal atoms loaded onto the surface. For instance, Ni is electrodeposited at −1.1 V vs. Ag/AgCl with a charge density of 0.2 mC cm^−2^ (hereinafter geometric area), which corresponds to ∼1.0 nmol cm^−2^, leading to ~6.2 atoms per nm^2^. The spontaneous oxidation of Ni in air generates H-NiO_x_ (Supplementary Fig. 3).

**Fig. 1:**
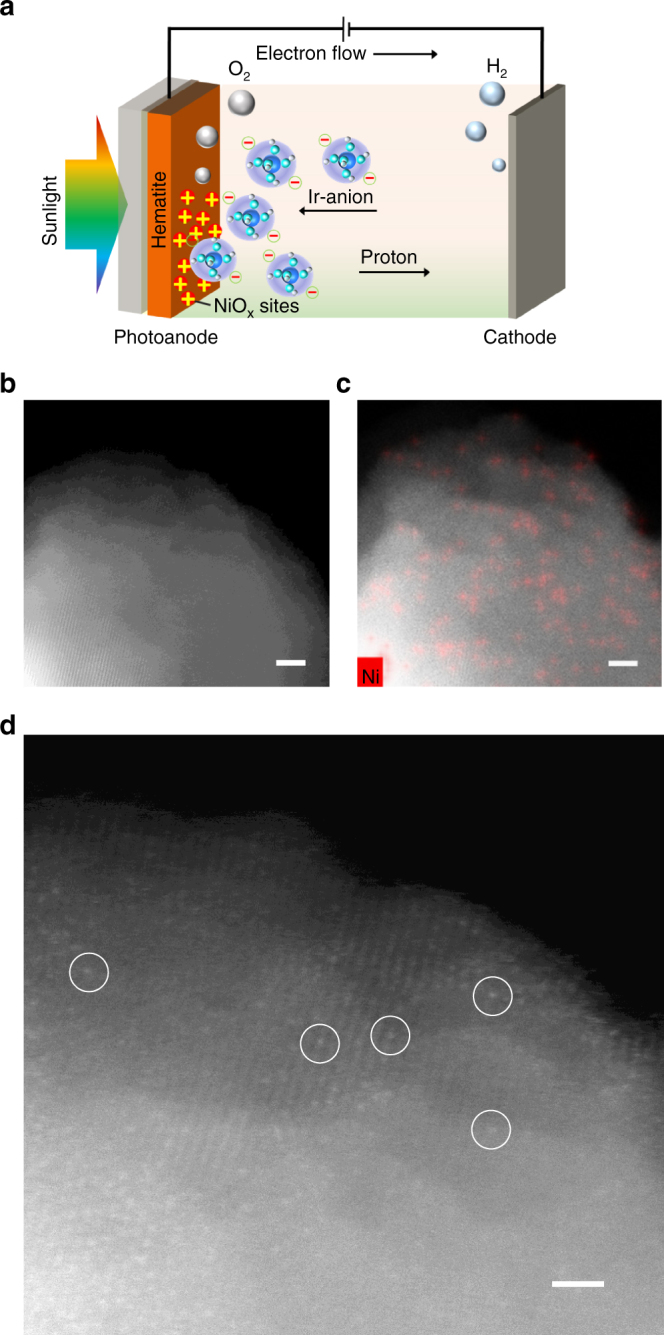
Synthesis and HAADF STEM images. **a** Schematic illustration of the preparation process at 1.23 *V*_RHE_ under AM 1.5 illumination in an electrolyte containing 1.0 M NaOH and 1.0 µM Ir-anion. **b** High-resolution HAADF STEM image of the hematite-supported catalyst and **c** Ni EDX mapping. Scale bar, 2 nm. **d** Isolated Ir atoms exemplarily indicated by circles are dispersed on the hematite. Scale bar, 1 nm

Unlike in the established methods for molecular catalyst loading, where pre-anchoring is required^18^, the soluble Ir-anion couples to H-NiO_x_ sites in situ during the OER at 1.23 *V*_RHE_ under AM 1.5 illumination in a photoelectrochemical (PEC) cell (Fig. 1a and Supplementary Fig. 4). The photo-excited holes reach the surface where the positive charges generate an electric field that attracts the negatively charged Ir-anions. When the measured photocurrent plateau is reached, we assume that the Ir-anion approaches an adsorption/desorption equilibrium, after which the loading of Ir on hematite reaches 0.3–1.6 atoms per nm^2^ (9.5–51.0 ng cm^−2^_Ir_) (Supplementary Note 1). Note that the non-adsorbed Ir-anion in the electrolyte is not consumed and thus recyclable.

### Site structure and catalysis

To identify the atomically dispersed site motifs, the dispersion of the resulting supported H-NiO_x_/Ir-anion hybrid was characterized by scanning transmission electron microscopy (STEM) in combination with energy-dispersive X-ray spectroscopy (EDX). Individual Ir atoms were imaged using atomic resolution high-angle annular dark field (HAADF) STEM. This technique offers *Z*-contrast conditions, i.e., the image intensity is roughly proportional to *Z* ^1.6^–*Z* ^1.9^, where *Z* is the atomic number of the present element (see Methods section). Therefore, the image contrast of Ir (*Z* = 77) strongly exceeds that of Fe (*Z* = 26) and Ni (*Z* = 28), which allows imaging of individual Ir atoms on a selected thin hematite region. Individual Ir atoms (exemplarily indicated by circles) are clearly separate on the hematite surface and show no indication of agglomeration or clustering in Fig. 1d in contrast to the Ir-free images (Supplementary Fig. 5). Figure 1c is a composite of an HAADF STEM image and a Ni EDX map that shows a homogeneous dispersion of Ni on the hematite substrate (Fig. 1b).

To assess how the mass transfer of Ir-anion influences the OER activity, two methods were carried out to prepare the Ir-anion-containing electrolytes (Supplementary Fig. 6). The first test was performed in 10 mL of homogeneous electrolyte containing 1.0 M NaOH and 1.0 µM Ir-anion (10 nmol Ir in total). The photocurrent density takes >1000 s to reach the plateau. The second method consisted of dripping an identical amount of Ir-anion solution into the 1.0 M NaOH at a close distance, about 0.5 cm away from the H-NiO_x_ electrode. The photocurrent increased suddenly to a maximum value and then started to decrease slowly as the momentary high concentration of Ir-anion close to the H-NiO_x_ electrode gradually diluted to a homogeneous electrolyte. This result indicates that the adsorption/desorption equilibrium is concentration-dependent and that only adsorbed Ir-anions can take part in the OER.

**Fig. 2:**
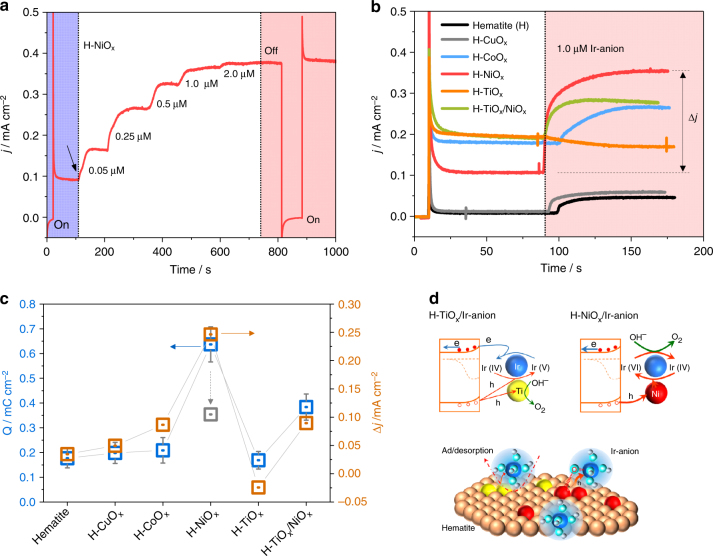
Photoelectrochemical tests. **a** OER activity of the H-NiO_x_/Ir-anion with a stepwise increase of Ir-anion solution concentration. **b** MO_x_-modified hematite exhibits significant activity difference (Δ*j*) upon dripping the Ir-anion-containing solution. **c** The correlation between the hole accumulation capacity of H-MO_x_ before the addition of Ir-anion (Supplementary Fig. 9) and the activity difference Δ*j* after the introduction of 1.0 µM Ir-anion relative to the Ir-anion-free electrolytes. The gray arrow shows the decreased capacity of H-NiO_x_ from 0.64 to 0.35 mC cm^−2^ (gray square) after the introduction of Ir-anion to the electrolyte. Error bars represent the standard deviation. **d** Proposed reaction paths explaining the different roles of TiO_x_ and NiO_x_ for the OER

**Fig. 3:**
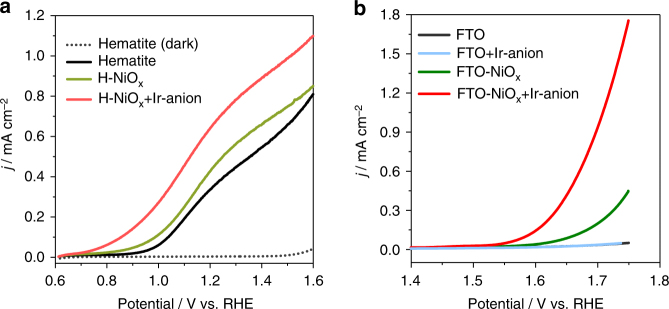
Catalytic *j*–*V* activity. **a** Linear sweep voltammograms (LSV) on Sn-doped hematite photoelectrodes under AM 1.5 irradiation and **b** dark FTO electrode at 10 mV s^−1^ in 1.0 M NaOH

Next, we evaluated the adsorption limitation of Ir-anions to the H-NiO_x_, with a fixed Ni loading of ∼1.0 nmol cm^−2^. Different concentrations of Ir-anion were chosen to maximize the catalyst’s activity. The H-NiO_x_ photoanode in 1.0 M NaOH at 1.23 *V*_RHE_ exhibits a steady-state photocurrent density of 0.09 ± 0.018 mA cm^−2^ upon switching on the light (Fig. 2a, blue region). After about 100 s, 5 µL of pre-prepared Ir-anion solution (1.0 M NaOH containing 0.1 mM Ir-anion) was dripped into the 10 mL 1.0 M NaOH electrolyte for the formation of a 0.05 µM Ir-anion solution. The photocurrent density increased to 0.17 mA cm^−2^ and then stabilized. A stepwise increase of Ir-anion concentration further enhances the photocurrent density until the Ir-anion concentration reaches about 2.0 µM (Fig. 2a, white region). This result is consistent with the variation of the surface Ir loading, where the surface Ir/Ni ratio increases slowly above 2.0 µM Ir-anion concentration (Supplementary Fig. 3). Ir-anion solution of 1.0 µM represents a good balance of cost-efficiency and high activity and was therefore selected for the following experiments. A chopped light illumination of the red region of Fig. 2a confirms that the enhanced photocurrent density resulted from light irradiation. This activity enhancement of Ir-anion on H-NiO_x_ relative to bare hematite agrees with the OER polarization curves (Supplementary Fig. 7). A decent activity enhancement was observed after the introduction of Ni/Ir hybrid catalyst on Sn-doped hematite. A considerable increase of the photocurrent density and a significant negative shift ~200 mV of the onset potential was observed, indicating increased reaction kinetics for water oxidation in Fig. 3a. The enhanced catalytic OER activity was confirmed on a dark F-doped SnO_2_ (FTO) electrode in Fig. 3b.

### Reaction mechanism

To uncover the interplay between NiO_x_ and Ir-anions and to understand the role of NiO_x_ sites, we screened a series of MO_x_ loadings with different metal ions (Fig. 2b). After dripping of an Ir-anion solution under stirring, an immediate and distinct photocurrent density increase was recorded for H-NiO_x_ but only a slight enhancement was tracked for pristine hematite, H-CuO_x_, and H-CoO_x_ (Fig. 2b, marked with pale color). In contrast, after addition of the Ir-anions, H-TiO_x_ shows a detrimental effect on OER (the surface coverage of TiO_x_ on the hematite is shown in Supplementary Fig. 5—this TiO_2_ sublayer formed by atomic layer deposition (ALD) is amorphous and has electronic defects that allow hole conduction^25^). Similar photocurrent decays were previously reported on mesoporous TiO_2_^23^ and W-doped BiVO_4_ photoanodes^24^, respectively. A poisoning mechanism was proposed in which the reversible adsorption/desorption of Ir-anion short-circuits the photoanode redox cycle at the interface between the semiconductor and the electrode FTO back contact^23^.

In the present study, the hematite semiconductor retains the same surface texture and the exposed FTO has no contribution to the activity of Ir-anion (Fig. 3b). The only difference leading to the activity difference is the decorated MO_x_ sites on the hematite surfaces, implying a strong dependence of the catalytic behaviors of Ir-anion on the surface properties of H-MO_x_ (Supplementary Fig. 8). The cluster-sized MO_x_ sites could be hydroxylated and amorphized under turnover in alkaline solution^26,27^. Notably, although TiO_x_ reduces the OER activity of the Ir-anions, the deposition of NiO_x_ onto H-TiO_x_ for the formation of an H-TiO_x_/NiO_x_ surface significantly increases the photocurrent again (Fig. 2b). This is direct evidence for a synergistic enhancement effect between the NiO_x_ and the Ir-anions.

Increased kinetics for OER requires a catalyst with an efficient surface hole density as shown in previous studies^28–30^. To assess the hole accumulation on MO_x_ sites, we used a transient photocurrent surface charging–discharging method to evaluate the density of accumulated holes under steady-state conditions during the OER at 1.23 *V*_RHE_. As soon as the photocurrent reaches the steady state, the light was switched off to record the cathodic current overshoot, owing to the continuing flux of electrons to the surface sites recombining with remaining holes (Supplementary Fig. 9). The number of the remaining holes describes the hole accumulation capacity of H-MO_x_ (Fig. 2c, open blue square), which is in good agreement with the calculated photocurrent density differences (*∆j*) of the H-MO_x_ photoanodes (Fig. 2c, open yellow square) derived from Fig. 2b. It is evident that the H-NiO_x_ sites exhibit much higher hole accumulation capacity relative to H-TiO_x_, thus promoting the four-electron OER process.

Before the accumulated holes can translocate to the soluble Ir-anions, the latter have to be bound to the semiconductor surface long enough to allow the photo-excited surface holes to continuously transfer to the Ir sites for catalysis^31–33^. The in situ formation of at least one “Fe/M–O–Ir” bond allows this continuous hole transfer to the Ir center. H-TiO_x_ is thermodynamically more stable and needs a higher overpotential for the hydroxylation by water as required for the formation of a “Fe/Ti–O–Ir” bond^34,35^. This makes the modified surface less efficient for transferring four holes to the Ir center for generating one oxygen molecule. Consequently, the Ir-anion is not able to catalyze the OER in the case of TiO_x_ but short-circuits the redox cycle, which was reflected in a decrease of the photocurrent (Fig. 2d, left). In contrast, the formation of “Fe/Ni–O–Ir” is favorable, which is consistent with the Ir-Ni binary surface^35^, allowing an instant immobilization of Ir-anion (Fig. 2d, bottom). To roughly quantify the possible ratio between NiO_x_ and Ir-anion, different Ni loading was applied to 2 nm thickness of amorphous conformal TiO_x_-covered hematite (H-2 nm TiO_x_)—relieving the influence of pristine hematite FeO_x_ sites. The concentration of the Ir-anion was kept at 1.0 µM. The optimal OER activity was achieved when the amount of NiO_x_ was four or more times higher than the immobilized Ir anions based on the HAADF STEM images and X-ray photoelectron spectroscopy (XPS) analysis (see Methods section). Further increasing or decreasing the amount of NiO_x_ leads to decreased OER activity (Supplementary Fig. 10). Thus, we propose that one Ir-anion may bridge one or more surface sites and this process depends on the pH of the solution^35,36^.

**Fig. 4:**
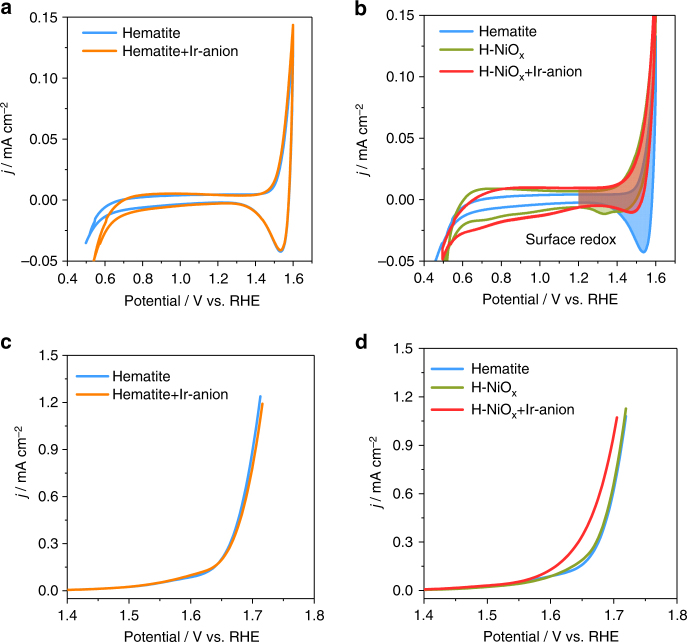
Charge transfer and activity enhancement. **a**, **b** Cyclic voltammetry at 50 mV s^−1^ and **c**, **d** linear sweep voltammetry at 10 mV s^−1^ on pristine hematite and H-NiO_x_ in 1.0 M NaOH in the dark. The concentration of Ir-anion is 1.0 µM

To evidence the hole transfer from the hematite surface to Ir-anion through the NiO_x_ site, cyclic voltammetry (CV) was performed on hematite and H-NiO_x_ in the dark (Fig. 4). Without NiO_x_ loading but in the presence of Ir-anions, the overlay of the CV curves of hematite and hematite/Ir-anion shows no detectable difference from 0.8 to 1.6 *V*_RHE_, implying no significant charge transfer between the hematite and the Ir-anions (Figs. 4a and 4c). When NiO_x_ was loaded on the hematite, the distinct cathodic peak at 1.55 *V*_RHE_ of surface iron species was suppressed (Supplementary Fig. 11), substituted by a new cathodic peak at 1.35 *V*_RHE_, arising from the Ni^III^/Ni^II^ redox couple on hematite substrate^37^. Once the Ir-anion was introduced to the electrolyte, the Ni reduction peak at 1.35 *V*_RHE_ decreased (Fig. 4b), concomitantly with a highly promoted catalytic activity peak (Fig. 4d), reflecting an efficient charge transfer from NiO_x_ to the Ir sites. This result is in agreement with the variation of charge transfer resistance across the catalyst/electrolyte interface evaluated by electrochemical impedance measurements (Supplementary Fig. 12). The pristine hematite did not show any significant resistance change after addition of the Ir-anions, whereas decoration of NiO_x_ on hematite decreased the resistance from 25.8 to 6.3 kΩ, and the subsequent addition of Ir-anions further decreased to 4.6 kΩ, implying the favorable charge transfer from NiO_x_ to Ir sites. Furthermore, the hole accumulation capacity of H-NiO_x_ decreased from 0.64 to 0.35 mC cm^−2^ after addition of the Ir-anions, (Fig. 2c, gray square), underlining that the Ni species transfers charge to the Ir centers^29^. These results confirm the continuous hole transfer from the hematite surface to the Ir-anions through the NiO_x_ sites. As an ideal molecular model, the NiO_x_ sites thus serve as “movable bridges” working within the potential region of OER (Fig. 2d, right), thereby improving the OER efficiency.

We verified that the evolved bubbles were indeed O_2_ using gas chromatography (GC). Based on the GC results and the measured photocurrents, the calculated faradaic yield of O_2_ is around 94% (Supplementary Fig. 13). The incident-photon-to-current efficiency (IPCE) was determined to confirm the promotion of hybrid Ir/Ni catalyst relative to the bare hematite (Supplementary Fig. 13). IPCE values match well the band gap of hematite and drop to zero at wavelengths beyond 600 nm.

### Stability test

Furthermore, we evaluated the stability of the H-NiO_x_ in strong alkaline electrolytes (1.0 and 4.0 M NaOH containing 1.0 μM Ir-anion). The H-NiO_x_ demonstrates extremely stable performance in both electrolytes, which makes it a strong candidate as a surface modifier for Ir-anions (Supplementary Fig. 14, gray line). After introducing the Ir-anions, an initial photocurrent increase at the beginning of the operation was attributed to the preferential adsorption of Ir-anions to the H-NiO_x_ surface. After this initial phase, the photocurrent density remains constant, indicating the superior stability (Supplementary Fig. 14, red line) over highly loaded IrO_x_ nanoparticles^15^. In 4.0 M NaOH, it takes a longer time period until the photocurrent reaches the plateau, probably due to the slower kinetics for the formation of “Fe/Ni–O–Ir” from the pH-dependent dehydroxylation of Ir-anion^36^.

To assess the stability of the adsorbed/bonded Ir-anions, the H-NiO_x_ photoanode and PEC cell were washed several times after 1 h of operation and freshly prepared NaOH was added to the PEC cell. The photocurrent density decayed slowly with increasing reaction time (Supplementary Fig. 14), suggesting a slow desorption process during the OER. To measure the reversibility of the Ir-anion adsorption/desorption, Ir-anions were again supplied to the electrolyte, which restored the photocurrent to the interrupted value, implying a self-healing feature (Supplementary Fig. 14a).

**Fig. 5:**
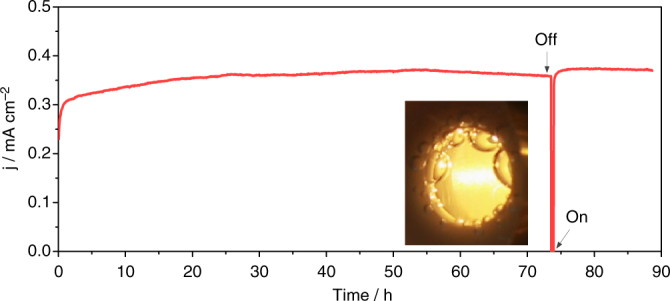
Stability test on H-NiO_x_. The chronopotentiometric curve obtained with the H-NiO_x_ photoelectrode in 4.0 M NaOH + 1.0 μM Ir-anion at 1.23 *V*_RHE_ under AM 1.5 illumination. A chopped light operation with 20 min interval was performed after 73 h of continuous stability test. The image of the photoanode window (inset) exhibits a bubble evolution/coverage on the surface of the Ni/Ir-decorated hematite photoanode

We also assessed the long-term performance stability in 4.0 M NaOH for >80 h of operation (Fig. 5). A continuous stability test of hybrid Ni/Ir sites over 73 h confirmed the high stability. In between, a chopped light operation with a 20 min interval was performed and the bubbles covering the electrode were removed by mechanically oscillating the electrolyte (inset of Fig. 5). Upon switching on the light, the photocurrent was restored and new bubbles formed gradually. This result corroborates that the hybrid Ni/Ir electrocatalyst satisfies the stability requirements of intermittent solar light illumination.

The rational design of a reaction condition-stable catalyst that selectively binds to the semiconductor surface sites under operating conditions increases the performance stability. The developed molecular-anchoring process provides a general photoelectrochemical route for synthesizing homogeneously and atomically dispersed, supported catalysts. Moreover, this study provides a promising path to explore new materials and strategies for combined heterogeneous/homogeneous electrocatalysis systems—an efficient and general strategy to employ other soluble molecular metal complexes^19^ or polyoxometalates^38^ on solid-state inorganic materials-modified semiconductors for solar-driven water splitting.

## Methods

### Synthesis of hematite and H-MO_x_

A nanostructured hematite was applied as a support for catalyst loading. It was prepared by hydrothermal deposition of β-FeOOH nanorods onto FTO glass (FTO, TEC 15 A 3.0 mm, XOP Física glass company) in an aqueous solution containing 0.15 M FeCl_3_·6H_2_O (Sigma-Aldrich), and 1.0 M NaNO_3_ (Sigma-Aldrich) at 95 °C for 1.5 h^39^. After the reaction, the as-prepared thin films were washed with ethanol/water several times and dried in flowing N_2_. Then the β-FeOOH thin films were thermally annealed at 600 °C for 2 h in the air leading to the formation of hematite structure. X-ray diffraction and Raman spectra imply the formation of hematite Fe_2_O_3_ (Supplementary Fig. 1). Scanning electron microscopy images show a rough surface that allows for catalyst loading (Supplementary Fig. 1).

The MO_x_ loading was achieved through a facile transient electroreduction of metal cations to metallic sites on hematite modified from the reported method^40^. This electrodeposition was implemented in a weak acidic electrolyte (pH = 4), which allows for a control of the amount of atoms deposited^40^, avoiding the spontaneous binding of cations on oxide semiconductor surfaces that happen in neutral and alkaline electrolytes. Controlling the charge going through the circuit manipulates the amount of metal atoms loaded onto the surface. For instance, the Ni is electroreduced at −1.1 V vs. Ag/AgCl with a charge density of 0.2 mC cm^−2^ (geometric area, hereinafter), taking about 0.1 s for the whole deposition (Supplementary Fig. 2). The amount of deposited Ni atoms on hematite corresponds to ∼1.0 nmol cm^−2^, leading to ~6.2 atoms per nm^2^. After electrodeposition, the spontaneous oxidation of Ni in air for ~3 days generates NiO_x_ sites. The main XPS peak positions of Ni_2p_ core level are at 873.5 and 856.0 eV (Supplementary Fig. 3); they are attributed to NiO_x_ sites. CuO_x_, CoO_x_, and NiO_x_ sites were equally prepared by this transient electrochemical process. The pristine hematite surface was considered as FeO_x_ sites. TiO_x_ has been deposited on hematite using an ALD system at 120 °C (PICSUN R-200). Tetrakis (dimethylamido)-titanium(IV) (99.999%, Sigma-Aldrich, kept at 85 °C) and H_2_O were used as Ti and O sources, respectively. Ti precursor was held in the chamber for 1.6 s under 150 sccm nitrogen flow, followed by a 6-s nitrogen purge. H_2_O was held for 0.1 s under 200 sccm nitrogen flow, followed by a 6-s nitrogen purge. TiO_x_ deposition has been calculated to be 0.50 Å per cycle, determined by ellipsometry on a silicon wafer with a native oxide layer.

### Synthesis of H-MO_x_/Ir-anion

Colorless [Ir(OH)_6_]^−2^ solution (0.1 mM) was prepared in 1.0 M NaOH by hydrolysis of K_2_IrCl_6_^36^. The Ir-anions were electrostatically adsorbed to the positively charged MO_x_ sites during the photoelectrochemical OER at 1.23 V vs. reversible hydrogen electrode (RHE) containing Ir-anions in NaOH electrolyte under AM 1.5 illumination.

### Materials characterizations

Material crystal phases of the β-FeOOH and the hematite were confirmed using X-ray diffraction and Raman. The X-ray diffraction studies were carried out in the range of scanning angle 20–70° using an X-ray diffractometer with Cu Kα radiation of wavelength 0.154060 nm (D8 Discover). A field emission scanning electron microscope (SEM Zeiss Supra 50 VP) was used to observe the surface morphology and estimate the surface texture. XPS was performed with a polychromatic MgKα (hν = 1253.6 eV) and a mono-chromatized AlKα (hν = 1486.6 eV) source in a modified Vacuum Generators ESCALAB 220 (*p* < 10^−9^ mbar)^41^. The energy scale was calibrated as described in this literature^42^. STEM studies were performed using an FEI Titan ChemiSTEM operated at 200 kV equipped with a Cs-probe corrector and a HAADF detector. This technique offers *Z*-contrast conditions, i.e., the image intensity is roughly proportional to *Z* ^1.6^–*Z* ^1.9^,where *Z* is the atomic number of the present element^43,44^. “*Z*-contrast” conditions were achieved using a probe semi-angle of 25 mrad and an inner collection angle of the detector of 70 mrad. Compositional maps were obtained with EDX using four large-solid-angle symmetrical Si drift detectors.

### Photoelectrochemical characterizations

Photoelectrochemical performance and the related electrochemical measurements were performed by using an SP-300 Potentiostat (Bio-logic). All measurements were made in alkaline electrolytes (1.0 or 4.0 M NaOH) in a custom-designed Cappuccino Teflon cell with a quartz window (Supplementary Fig. 4). A standard three-electrode setup was applied by using hematite or H-MO_x_ photoelectrodes as the working electrode and platinum gauze as the counter electrode. All the measured potentials are vs. RHE. A 300 W Xenon lamp (LOT-QuantumDesign GmbH, Germany) coupled to a filter (AM 1.5) was used to simulate solar light. The light power density was calibrated to 100 mW cm^−2^ (AM 1.5) with a Si reference solar cell (LOT-QuantumDesign GmbH, Germany).

### Data availability

The data that support the findings of this study are available on reasonable request from the corresponding author (C.C.).

## Electronic supplementary material

Supplementary Information

Peer Review File
