# Supplementary material for: Atomically dispersed hybrid nickel-iridium sites for photoelectrocatalysis
Source: Nat Commun. 2017 Nov 7;8:1341. doi: 10.1038/s41467-017-01545-w (PMC5677126; doi:10.1038/s41467-017-01545-w)
Supplement: Supplementary file 1 — Supplementary Information [file 41467_2017_1545_MOESM1_ESM.pdf]

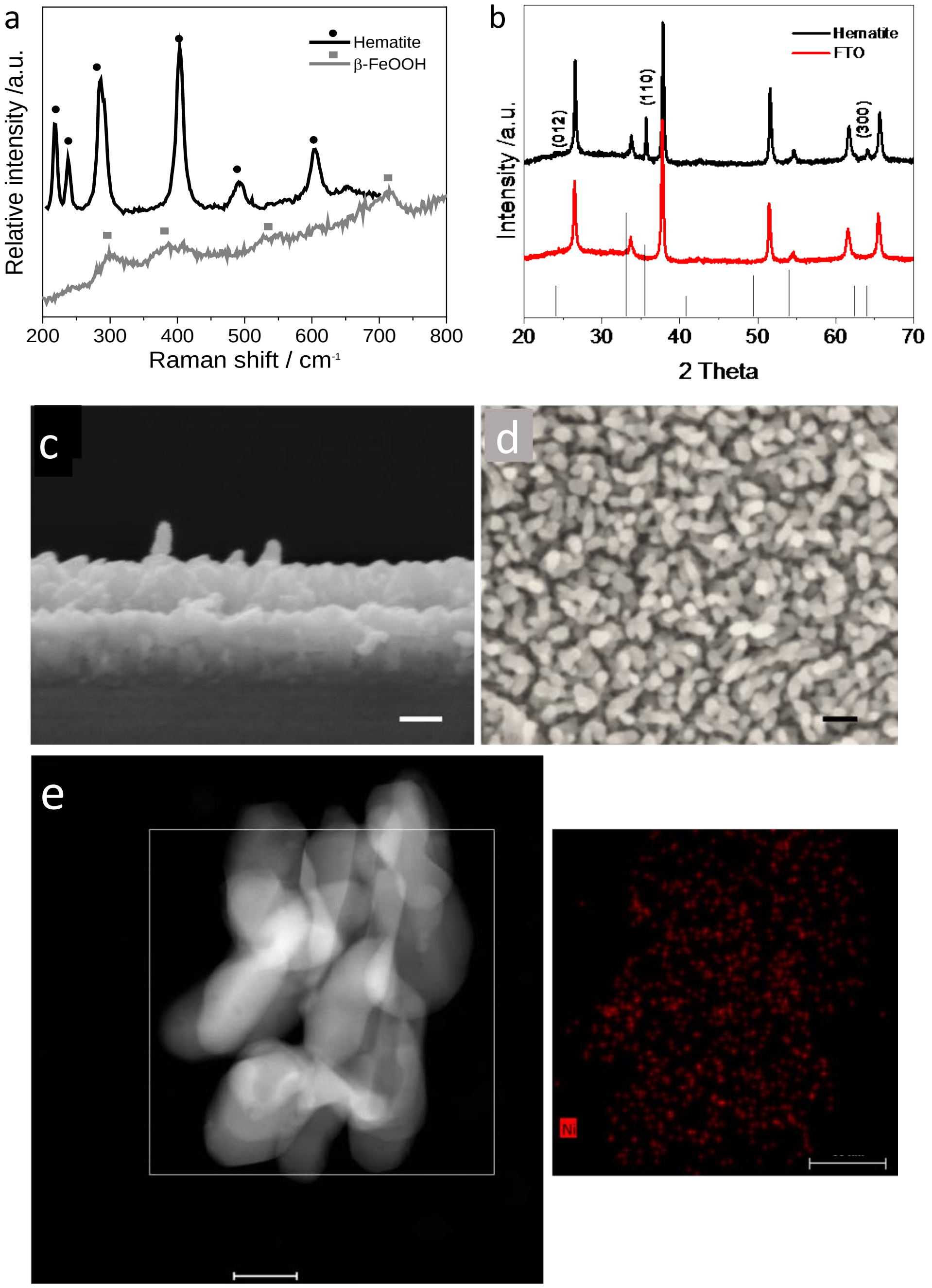

Supplementary Figure 1. Crystal phase and surface morphology of hematite. (a) Raman spectra of as-prepared β-FeOOH and hematite films, (b) X-ray diffraction of hematite film, and (c) cross-section and (d) surface morphology of the hematite film. Scale bar, 100 nm. (e) Ni distribution on the surface of hematite by elemental mapping. Scale bar of image, 50 nm. Scale bar of Ni map, 60 nm. High resolution image can be found in Figure 1c.

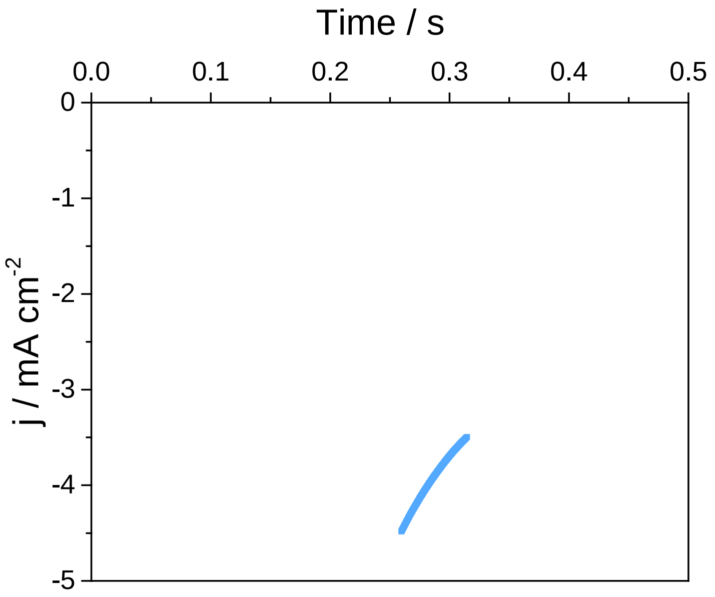

Supplementary Figure 2. A transient electro-reduction of Ni ions at -1.1 V versus Ag/AgCl with a charge density of 0.20 mC cm^{-2}. The whole deposition process takes around 0.1 seconds.

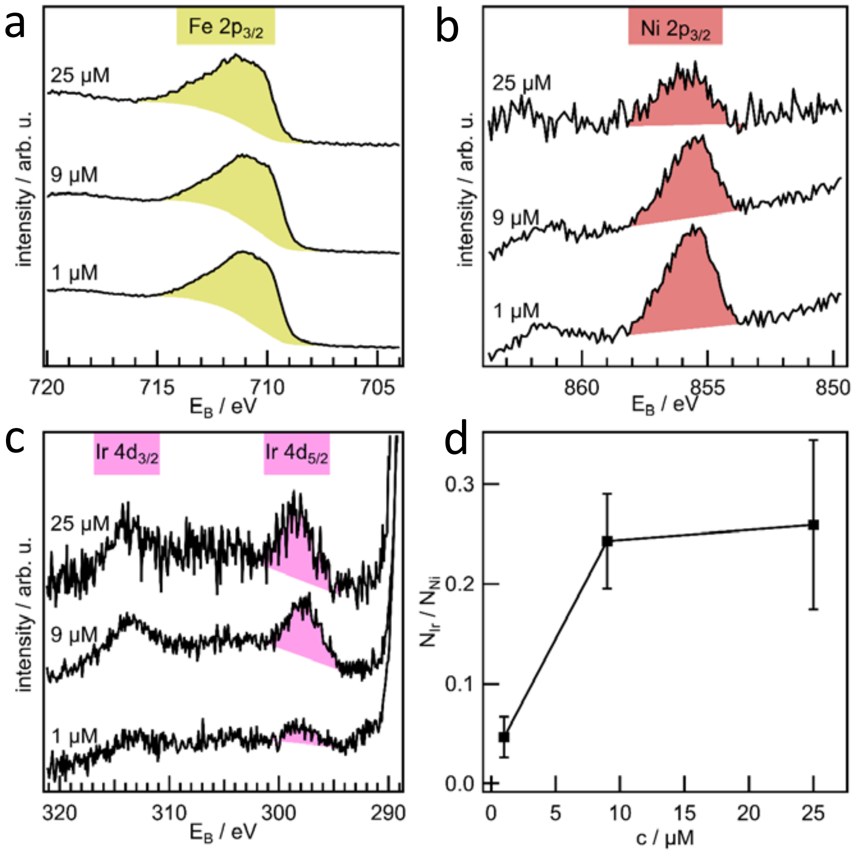

Supplementary Figure 3. XPS evaluation. (**a**) Fe $2p_{3/2}$, (**b**) Ni $2p_{3/2}$ and (**c**) Ir 4d corelevel spectra with different Ir concentrations in the solution. The shaded peak areas are used for calculating the loadings. (**d**) The ratio of Ir/Ni as a function of Ir solution concentration assuming Ni and Ir are homogeneously dispersed on the surface of hematite. Error bars represent standard deviation.

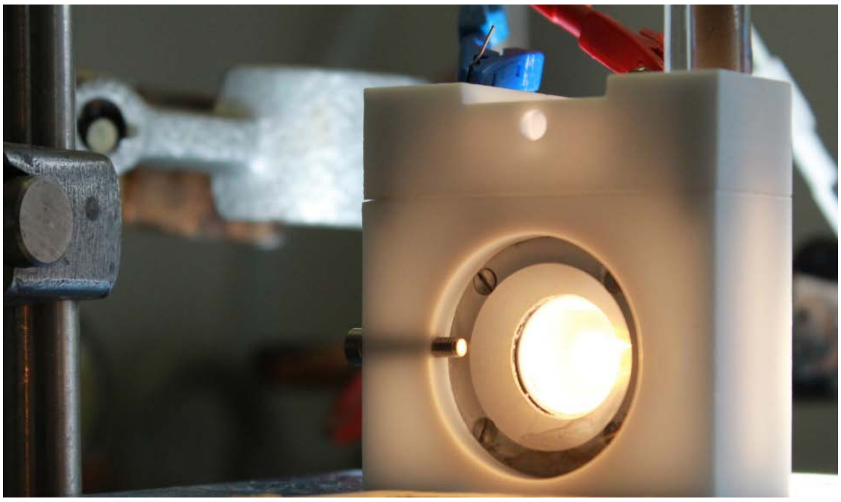

Supplementary Figure 4. The overview of the photoelectrochemical cell (PEC cell).

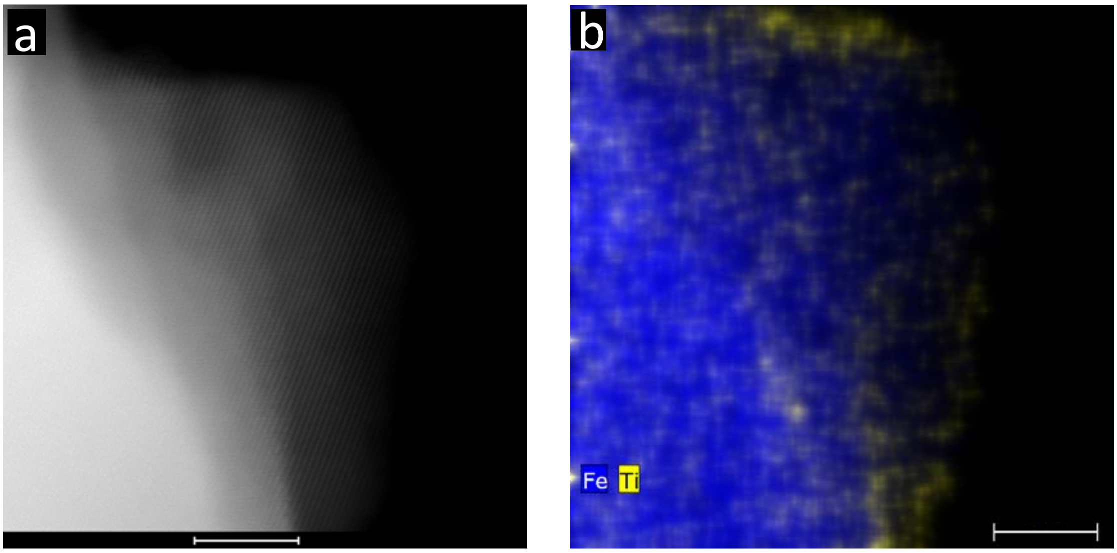

Supplementary Figure 5. Element mapping. **(a)** HAADF and **(b)** EDX mapping of Fe and Ti of H-TiO_x. Scale bar, 5 nm.

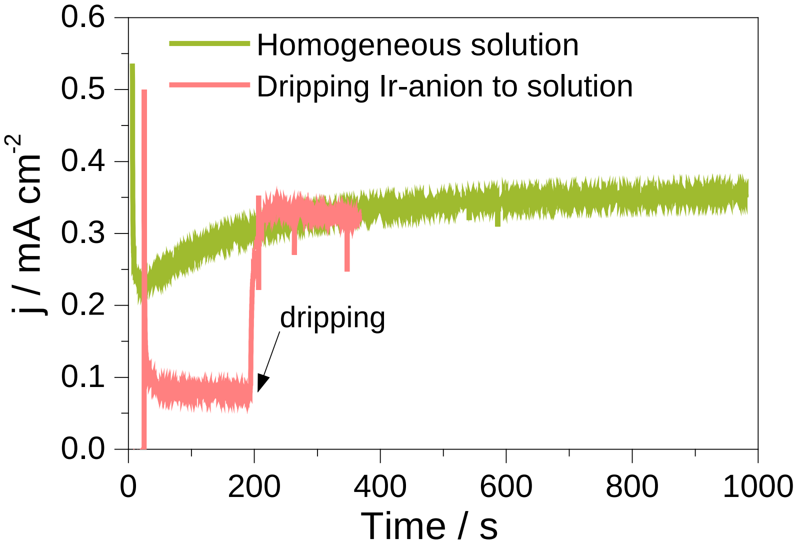

Supplementary Figure 6. Ir-anion decoration on the H-NiO$_x$. Two electrolytes were used for photoelectrochemical decoration of Ir-anions onto the H-NiO$_x$ at 1.23 V versus RHE under AM1.5 illumination. In contrast to a homogeneous electrolyte presenting gradual increase of the photocurrent with a locally lower Ir-anion concentration, the dripping route with an instant high concentration could cause surface adsorption but might loss adsorption selectivity around NiO$_x$ sites, thus leading to different photocurrent plateaus.

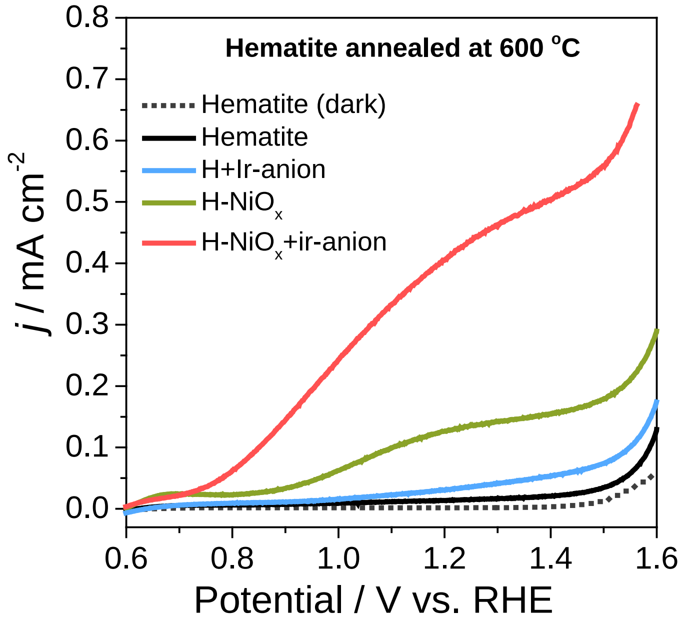

Supplementary Figure 7. Linear sweep voltammograms (LSV) on hematite photoelectrodes under AM1.5 irradiation at 10 mV/s in 1.0 M NaOH. The intrinsic hematite annealed at 600 ^{o}C for 2 hours exhibited photoactivity about 15 μA cm^{-2}, in line with the reported value (10 μA cm^{-2})[1].

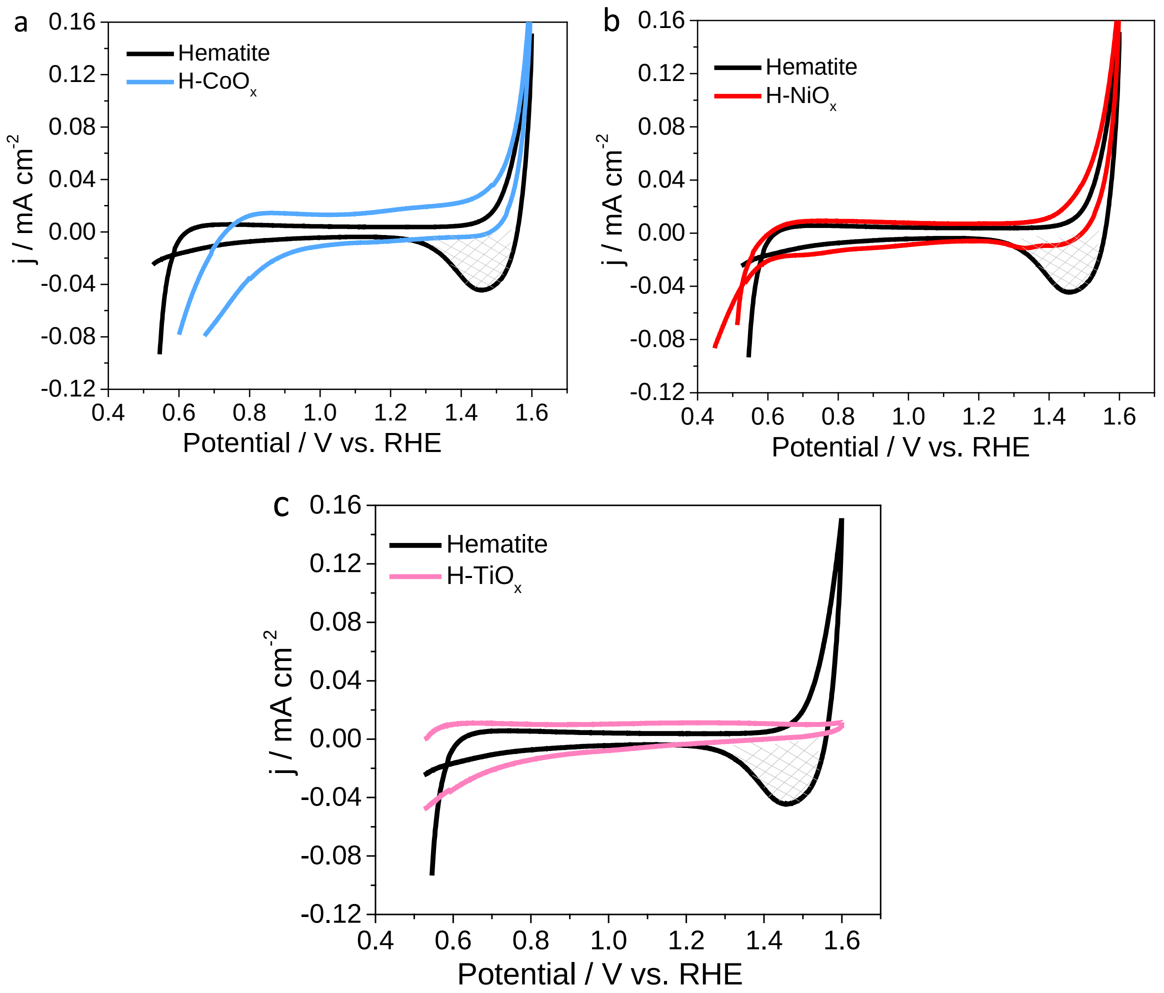

Supplementary Figure 8. Surface properties of the H-MO_x. (a) H-CoO_x, **(b)** H-NiO_x, and **(c)** H-TiO_x. The MO_x sites loaded on hematite vary the surface properties. After decoration of CoO_x sites, the redox peak of bare hematite surface was suppressed in the dark, implying that the CoO_x modified the surface redox couple. This argument is also the same in H-NiO_x. The TiO_x also inhibited the redox peak of hematite but with a catalytically inactive surface and thus served as a passivation layer the same as Ga_2O_3[2].

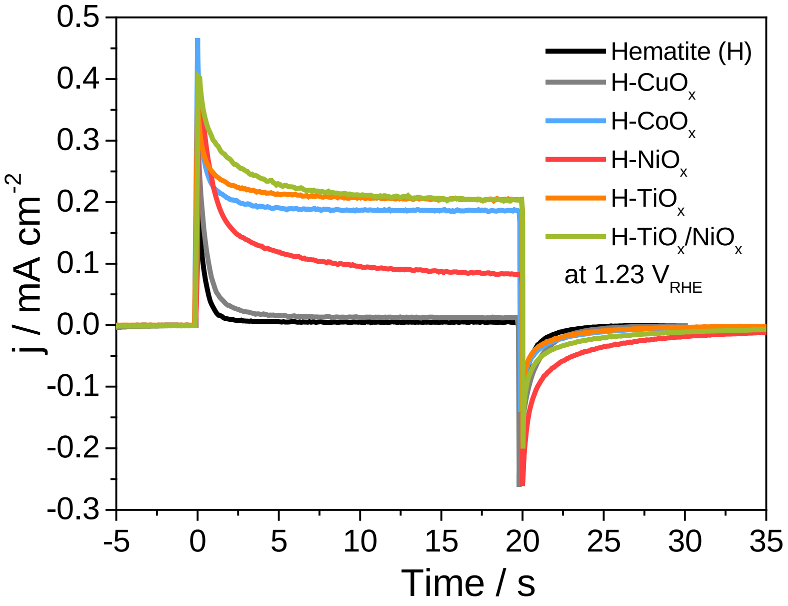

Supplementary Figure 9. Relationship between the surface hole accumulation capacity (Q) of H-MO_x and the photocurrent density enhancement (*Δj*). Considering an identical operation condition, a fixed electrochemical potential was chosen for evaluation. Surface hole density at the steady state during OER in the absence of Ir-anions has been used to evaluate the capacity of hole accumulation. The lifetime of the holes on the hematite surface is on the order of 0.1−10 second but the holes in the space-charge region recombine within the picosecond-millisecond timescale[3, 4, 5].The fast recombination of holes in the space-charge region cannot be observed in photocurrent analyses because these electrons are not extracted. Thus, only the “long-lived” holes accumulated on the surface can be counted in the cathodic recombination photocurrent. The calculated *Q* can be found in Fig. 2C in the main text.

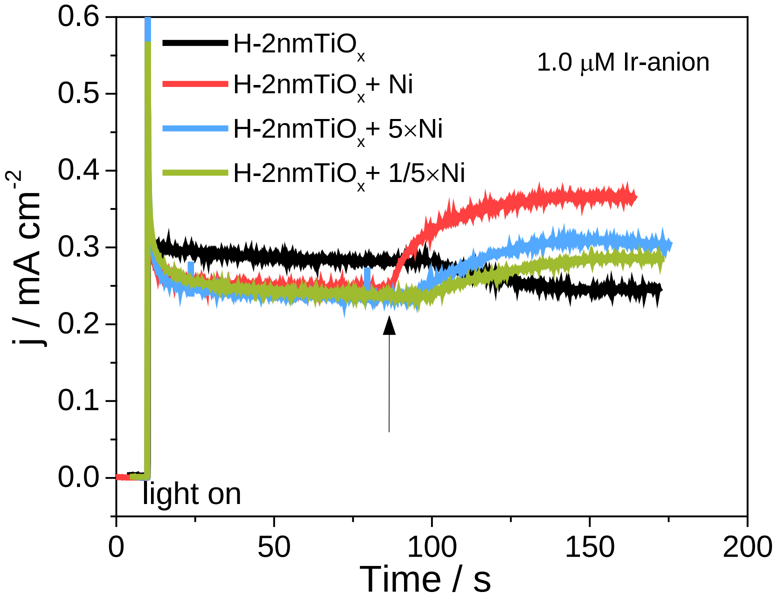

Supplementary Figure 10. Photoelectrochemical tests on H-2nmTiO_x and H-2nmTiO_x-NiO_x photoelectrodes. A photocurrent decay obtained without decoration of NiO_x in Ir-anion containing electrolyte again confirms that the TiO_x has a detrimental influence on the OER. The deposition of homogeneously dispersed NiO_x on H-2nmTiO_x decreases the steady state photocurrent probably due to the high capacity of hole accumulating but the low kinetics of water oxidation at NiO_x where serves as recombination sites[6].

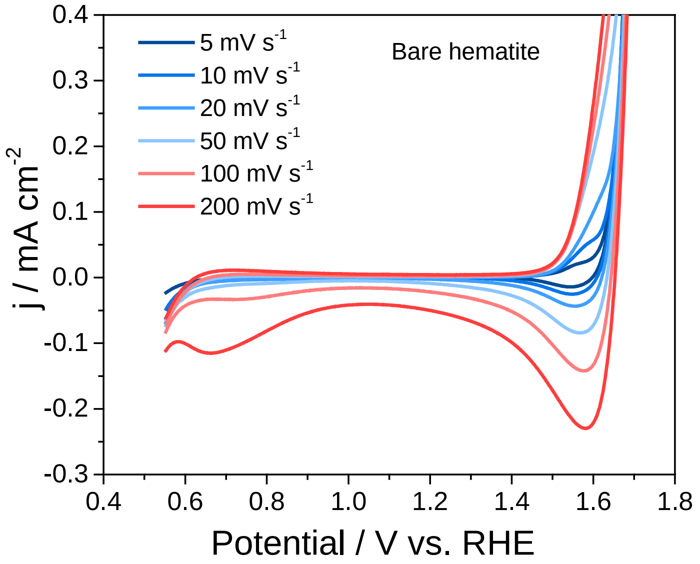

Supplementary Figure 11. Scan rate-dependent electrochemical tests of bare hematite in the dark in the potential region of 0.6-1.7 V vs RHE. The observed cathodic peaks are consistent with the results in the literature [7], which implies surface Fe redox species[8].

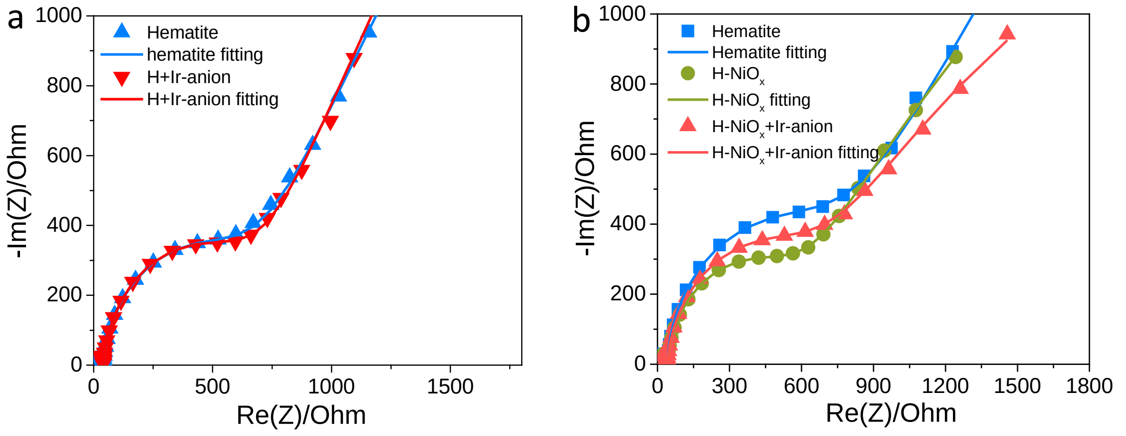

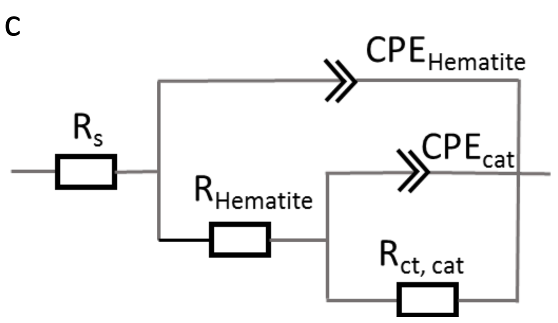

d

	Photoanodes	$R_{ct,cat}$ (Ω)
A	$Hematite_1$	25,107
	H_1/-Ir	21,760
B	$Hematite_2$	25,805
	H_2+NiO_x	6,346
	H_2+NiO_x/-Ir	4,564

Supplementary Figure 12. Electrochemical impedance on bare hematite and H-NiO$_x$. Electrochemical impedance was used to clarify the improved charge transfer resistance. It was measured at 0.0 V versus RHE under illumination and shown as Nyquist plots (**a** and **b**). The data was fitted to an equivalent circuit (**c**) containing constant phase elements of $CPE_{Hematite}$ and CPE_{cat} and charge transfer resistances ($R_{Hematite}$) from hematite to catalyst and ($R_{ct,cat}$) from the catalyst to redox couple in an electrolyte[9, 10]. The $R_{ct,cat}$ usually represents the charge behavior at catalyst/electrolyte interface. Charge transfer resistances extracted from the fittings are summarized in table **d**. The resistance $R_{ct,cat}$ decreased from 25.8 to 6.3 kΩ after loading NiO$_x$ and further decreased to 4.5 kΩ after the addition of Ir-anion indicates the favorable charge transfer across the interface, reflected as increased catalytic activity of the hybrid Ni/Ir catalyst. In contrast, without NiO$_x$ but in the presence of Ir-anions, we did not observe a significant decrease of charge transfer resistance. We performed the tests on two individual bare hematite samples, $hematite_1$ and $hematite_2$, which are basically identical.

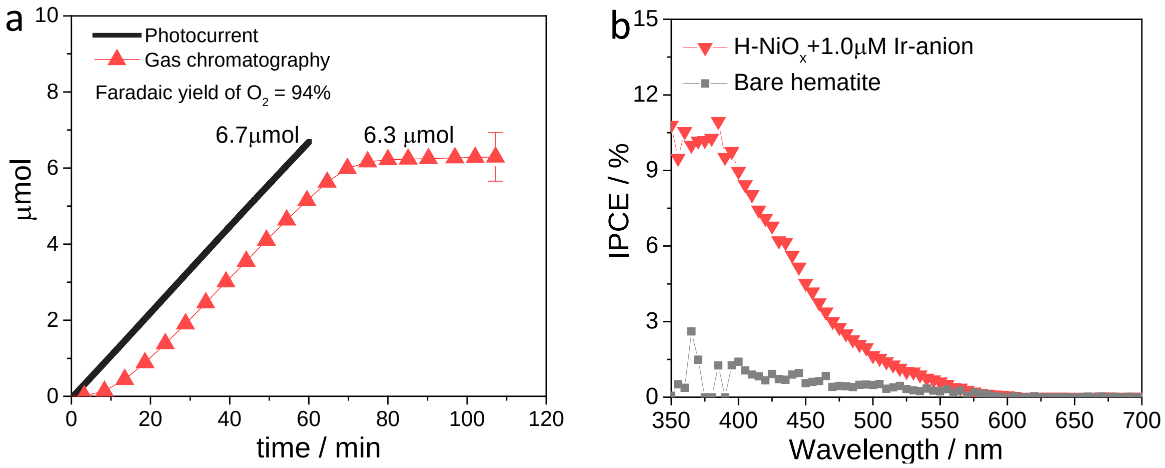

Supplementary Figure 13. Verification of O_2, faradaic yield, and the incident-photon-to-current efficiency (IPCE). (**a**) Verification of O_2 evolution and faradaic yield of O_2 through using gas chromatography (Bruker Corporation 450 GC). The test was performed after the photocurrent density reached the plateau in 1.0 M NaOH containing 1.0 μM Ir-anions at 1.23 V vs. RHE under illumination. The exposed electrode area to the electrolyte is not specified. The evolved O_2 gas during the 60 min was analyzed. The estimated faradaic yield of O_2 is ~94 %. Error bars represent standard deviation. Since the Ir sites are hydroxylated, there is no lattice oxygen binding to this Ir catalyst, in sharp contrast to core-shell structured IrO_x and mixed core-shell $NiIrO_x$[11, 12], where the catalytic activity of surface IrO_x is highly influenced by the subsurface composition and structure in the core region. (**b**) The IPCE was measured using a monochromatic light irradiation (LOT-QuantumDesign GmbH). Integration of the IPCE after loading of hybrid Ni/Ir catalyst yields 0.366 mA cm^{-2}.

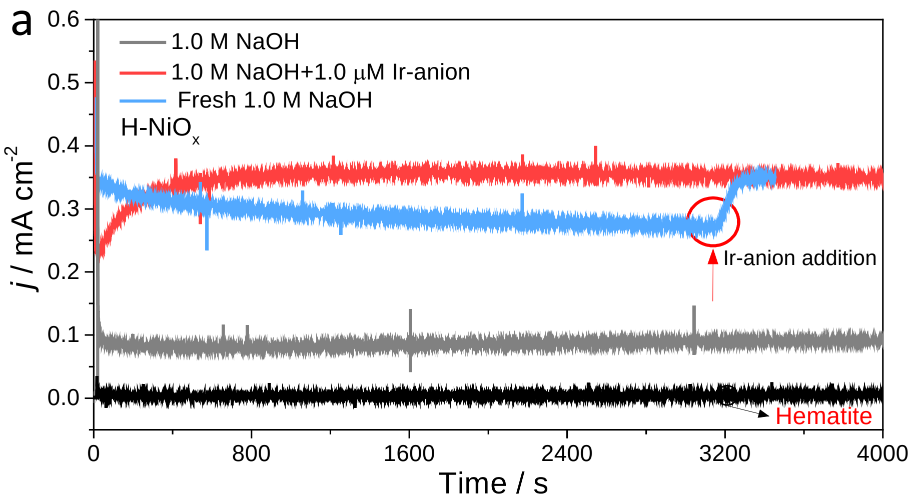

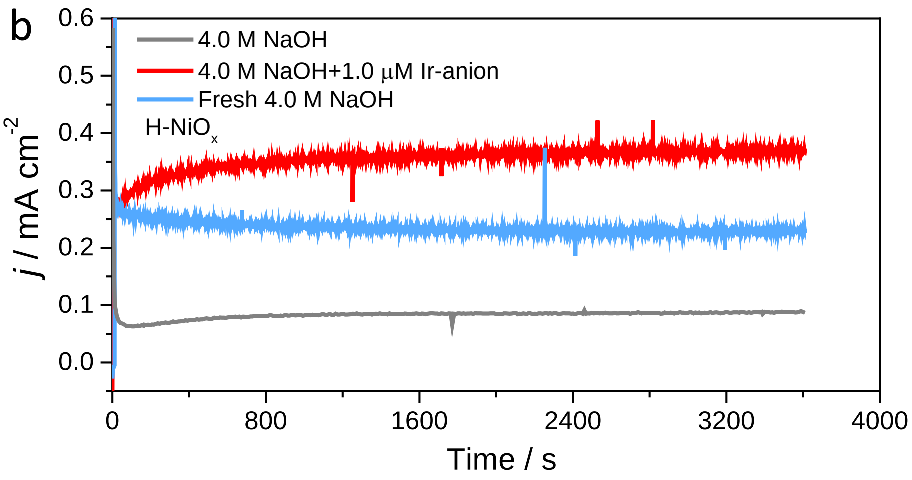

Supplementary Figure 14. The chronopotentiometric curves of H-NiO$_x$ in strong alkaline electrolytes. (a) 1.0 M and **(b)** 4.0 M NaOH. The chronopotentiometric curve with black color in **(a)** shows the stability of bare hematite. The red arrow in (**a**) shows the refilling of Ir-anions.

Supplementary Note 1

Estimated loadings of Ir and turnover frequency (TOF) calculations

The accurate estimate of Ir loading is challenging owing to the following reasons: first of all, the adsorption/desorption of Ir-anions is dynamic. After the photocurrent density reached the plateau, where we assume that adsorption/desorption was close to the equilibrium. We only considered the loading of Ir-anions after this time point. Second, the estimate of Ir loading by instruments is difficult owing to its extremely low amount.

We indirectly evaluated the Ir loading with respect to Ni loading. The electrodeposited Ni is ~6.2 atoms nm^{-2}. We estimated the Ir/Ni ratio by quantitative XPS analysis of the Ni $2p_{3/2}$ and the Ir $4d_{5/2}$ peaks. The corelevel were recorded with a monochromatic AlKα source (Supplementary Figures 3a-c). The N_{Ir}/N_{Ni} ratio is calculated according to:

$N_{Ir}/N_{Ni} = (I_{Ir}/\sigma_{Ir}) \times (\sigma_{Ni}/I_{Ni})$

Where Ix is the measured intensity of the Ni $2p_{3/2}$ or Ir $4d_{5/2}$ peak, σ_X is the photoionization cross section[13],

The Ir/Ni ratio as a function of solution Ir concentration is presented in Supplementary Figure 3d.The adsorbed Ir-anions approach to saturation at the 25μM Ir solution concentration.At 1.0μM andbased on the Ni loading and the Ir/Ni ratio, Ir loading is ~0.3atoms nm^{-2} (~0.048 nmol cm^{-2}), which is used for turnover frequency calculation with an upper limit (TOF_{up}).At 25μM and based on the Ni loading and the Ir/Ni ratio, Ir loading is ~1.6atoms nm^{-2} (~0.257 nmol cm^{-2}), which is used for turnover frequency calculation with a lower-limit (TOF_{low}).

Based on the estimated Faradaic efficiency of 94 % and assuming that every adsorbed Ir atom takes part in the OER, then the TOF value was calculated from the equation[14].

$TOF_{low}=(\Delta j \times A)/(4\times F\times n)=(0.25*0.2826*10^{-3}*94\%)/(4*96485.3*0.257*10^{-9}*0.2826)=2.4\ s^{-1}$

$TOF_{up}=(\Delta j \times A)/(4\times F\times n)=(0.25*0.2826*10^{-3}*94\%)/(4*96485.3*0.048*10^{-9}*0.2826)=12.7\ s^{-1}$

where Δj is the photocurrent density difference under the steady state (Δj, ~0.25 $mAcm^{-2}$) at 1.23 V versus RHE. A (0.2826 cm^2) is the geometric area exposed to the electrolyte. F is the Faraday constant (96485.3 C mol^{-1}). n is the atomic loading of Ir on the surface evaluated by electrochemistry, XPS, and STEM EDX analysis.

Supplementary Note 2

The reasons that the intrinsic hematite was selected as a platform

First of all, at present n-type hematite photoanodes which are able to exhibit enhanced photocurrent densities have to be intentionally doped through either high-temperature-diffusion induced doping of Sn from FTO layer or extrinsic doping. However, the doped metals could diffuse to the surface of hematite with the same potential of binding to the soluble Ir-anions as the MO_x sites do. They may thereby serve as active sites interfering the evaluation of Ir-anion loading and influencing the final water oxidation performance. Thus, to rule out this factor, the intrinsic hematite has simplified surface parameter and thus selected as an ideal platform. Secondly, to carefully evaluate the hole accumulation capability of transition metal oxides and exhibit their interactions with soluble molecular catalysts for water oxidation, the intrinsic hematite photoanode with a much less light-excited hole density on the surface is favorable as the accumulated hole signal by transition metal oxide is able to be electrochemically amplified under pretty low charge background. Low charge density means high charge resolution so that we are able to exhibit the hole accumulation behavior of cluster-sized transition metal oxides. Concerning a further extension of catalysts on highly doped semiconductors[15, 16], Ni/Ir loaded on Sn-doped hematite demonstrates a decent photocurrent enhancement and a significant negative shift ~200 mV of the onset potential, suggesting the viable application as a promising catalyst (Figure 3).

Supplementary References

1. Kay A, Cesar I, Gratzel M. New benchmark for water photooxidation by nanostructured alpha-Fe_2O_3 films. *J Am Chem Soc* **128**, 15714-15721 (2006).

2. Barroso M*, et al.* Dynamics of photogenerated holes in surface modified alpha-Fe_2O_3 photoanodes for solar water splitting. *Proc Natl Acad Sci U S A* **109**, 15640-15645 (2012).

3. Le Formal F, Pendlebury SR, Cornuz M, Tilley SD, Grätzel M, Durrant JR. Back electron–hole recombination in hematite photoanodes for water splitting. *J Am Chem Soc*, (2014).

4. Appavoo K, Liu MZ, Black CT, Sfeir MY. Quantifying bulk and surface recombination processes in nanostructured water splitting photocatalysts via in situ ultrafast spectroscopy. *Nano Lett* **15**, 1076-1082 (2015).

5. Peter LM, Wijayantha KGU, Tahir AA. Kinetics of light-driven oxygen evolution at alpha-Fe_2O_3 electrodes. *Faraday Discuss* **155**, 309-322 (2012).

6. Ma Y, Kafizas A, Pendlebury SR, Le Formal F, Durrant JR. Photoinduced absorption spectroscopy of CoPi on $BiVO_4$: the function of CoPi during water oxidation. *Adv Funct Mater* **26**, 4951-4960 (2016).

7. Moir J*, et al.* Activation of ultrathin films of hematite for photoelectrochemical water splitting via H_2 treatment. *ChemSusChem* **8**, 1557-1567 (2015).

8. Jang J-W*, et al.* Enabling unassisted solar water splitting by iron oxide and silicon. *Nat Commun* **6**, (2015).

9. Klahr B, Gimenez S, Fabregat-Santiago F, Hamann T, Bisquert J. Water oxidation at hematite photoelectrodes: the role of surface states. *J Am Chem Soc* **134**, 4294-4302 (2012).

10. Ding Q*, et al.* Efficient photoelectrochemical hydrogen generation using heterostructures of Si and chemically exfoliated metallic MoS_2. *J Am Chem Soc* **136**, 8504-8507 (2014).

11. Nong HN, Gan L, Willinger E, Teschner D, Strasser P. IrO_x core-shell nanocatalysts for cost- and energy-efficient electrochemical water splitting. *Chem Sci* **5**, 2955-2963 (2014).

12. Moghaddam RB, Wang C, Sorge JB, Brett MJ, Bergens SH. Easily prepared, high activity Ir-Ni oxide catalysts for water oxidation. *Electrochem Commun* **60**, 109-112 (2015).

13. Scofield JH. Hartree-Slater subshell photoionization cross-sections at 1254 and 1487 eV. *J Electron Spectrosc Relat Phenom* **8**, 129-137 (1976).

14. Zhang B*, et al.* Homogeneously dispersed, multimetal oxygen-evolving catalysts. *Science* **352**, 333-337 (2016).

15. Du C*, et al.* Hematite-based water splitting with low turn-on voltages. *Angew Chem Int Ed* **52**, 12692-12695 (2013).

16. Kim JY*, et al.* Single-crystalline, wormlike hematite photoanodes for efficient solar water splitting. *Sci Rep* **3**, 2681-2681 (2013).
