# Supplementary material for: Atomically dispersed hybrid nickel-iridium sites for photoelectrocatalysis
Source: Nat Commun. 2017 Nov 7;8:1341. doi: 10.1038/s41467-017-01545-w (PMC5677126; doi:10.1038/s41467-017-01545-w)
Supplement: Supplementary file 2 — Peer Review File [file 41467_2017_1545_MOESM2_ESM.pdf]

Editorial Note: this manuscript has been previously reviewed at another journal that is not operating a transparent peer review scheme. This document only contains reviewer comments and rebuttal letters for versions considered at Nature Communications.

Reviewer #1 (Remarks to the Author):

In the revised manuscript and response letter, the authors have fully addressed the concerns. I recommend it to be accepted for publication.

Reviewer #2 (Remarks to the Author):

The authors adequately addressed my concerns, and I recommend to publish the manuscript as is.

Reviewers Comments:

REVIEWERS' COMMENTS:

Reviewer #1 (Remarks to the Author):

In the revised manuscript and response letter, the authors have fully addressed the concerns. I recommend it to be accepted for publication.

Reviewer #2 (Remarks to the Author):

The authors adequately addressed my concerns, and I recommend to publish the manuscript as is.

Reply:

We truly appreciate Reviewer #1 and Reviewer #2 for publication recommendation of this work.
